# Patients’ Use of Mobile Health for Self-management of Knee Osteoarthritis: Results of a 6-Week Pilot Study

**DOI:** 10.2196/30495

**Published:** 2021-11-25

**Authors:** Brittany Shewchuk, Lee A Green, Tanya Barber, Jean Miller, Sylvia Teare, Denise Campbell-Scherer, Kelly J Mrklas, Linda C Li, Nancy Marlett, Tracy Wasylak, Elena Lopatina, Deirdre McCaughey, Deborah A Marshall

**Affiliations:** 1 Department of Community Health Sciences Cumming School of Medicine University of Calgary Calgary, AB Canada; 2 Department of Family Medicine University of Alberta Edmonton, AB Canada; 3 Enhancing Alberta Primary Care Research Networks Department of Family Medicine University of Alberta Edmonton, AB Canada; 4 O'Brien Institute for Public Health University of Calgary Calgary, AB Canada; 5 Department of Family Medicine Faculty of Medicine and Dentistry University of Alberta Edmonton, AB Canada; 6 Office of Lifelong Learning and Physician Learning Program Faculty of Medicine and Dentistry Edmonton, AB Canada; 7 Strategic Clinical Networks System Innovation and Programs Alberta Health Services Calgary, AB Canada; 8 Department of Physical Therapy University of British Columbia Vancouver, BC Canada; 9 Patient and Community Engagement Research Unit O'Brien Institute for Public Health Calgary, AB Canada; 10 Community Rehabilitation and Disability Studies Cumming School of Medicine University of Calgary Calgary, AB Canada; 11 Strategic Clinical Networks Alberta Health Services Calgary, AB Canada; 12 Faculty of Nursing University of Calgary Calgary, AB Canada; 13 Department of Community Health Sciences Faculty of Medicine University of Calgary Calgary, AB Canada

**Keywords:** mobile health, mHealth, app, self-management, osteoarthritis

## Abstract

**Background:**

In a previous study, a prototype mobile health (mHealth) app was co-designed with patients, family physicians, and researchers to enhance self-management and optimize conservative management for patients with mild to moderate knee osteoarthritis (OA).

**Objective:**

This study aims to evaluate the overall usability, quality, and effectiveness of the mHealth app prototype for aiding knee OA self-management from the perspectives of patients with OA and health care providers (HCPs).

**Methods:**

Using methods triangulation of qualitative and quantitative data, we conducted a pilot evaluation of an mHealth app prototype that was codeveloped with patients and HCPs. We recruited adult patients aged ≥20 years with early knee OA (n=18) who experienced knee pain on most days of the month at any time in the past and HCPs (n=7) to participate. In the qualitative assessment, patient and HCP perspectives were elicited on the likeability and usefulness of app features and functionalities and the perceived impact of the app on patient-HCP communication. The quantitative assessment involved evaluating the app using usability, quality, and effectiveness metrics. Patient baseline assessments included a semistructured interview and survey to gather demographics and assess the quality of life (European Quality-of-Life 5-Dimension 5-Level Questionnaire [EQ-5D-5L]) and patient activation (patient activation measure [PAM]). Following the 6-week usability trial period, a follow-up survey assessed patients’ perceptions of app usability and quality and longitudinal changes in quality of life and patient activation. Semistructured interviews and surveys were also conducted with HCPs (n=7) at baseline to evaluate the usability and quality of the app prototype.

**Results:**

Interviews with patients and HCPs revealed overall positive impressions of the app prototype features and functionalities related to likeability and usefulness. Between the baseline and follow-up patient assessments, the mean EQ-5D-5L scores improved from 0.77 to 0.67 (*P*=.04), and PAM scores increased from 80.4 to 87.9 (*P*=.01). Following the 6-week evaluation, patients reported a mean System Usability Scale (SUS) score of 57.8, indicating marginal acceptability according to SUS cutoffs. The mean number of goals set during the usability period was 2.47 (SD 3.08), and the mean number of activities completed for knee OA self-management during the study period was 22.2 (SD 17.8). Spearman rank correlation (*r_s_*) calculations revealed that the follow-up PAM scores were weakly correlated (*r_s_*=−0.32) with the number of goals achieved and the number (*r_s_*=0.19) of activities performed during the 6-week usability period. HCPs reported a mean SUS score of 39.1, indicating unacceptable usability.

**Conclusions:**

This evidence-based and patient-centered app prototype represents a potential use of mHealth for improving outcomes and enhancing conservative care by promoting patient activation and patient-HCP communication regarding OA management. However, future iterations of the app prototype are required to address the limitations related to usability and quality.

## Introduction

### Background

Osteoarthritis (OA) is the most common form of arthritis and a leading cause of disability worldwide [1]. Current management strategies for knee OA largely target symptom control with the primary aim of reducing pain, improving function, and delaying the need for surgical intervention [2]. There are currently no disease-modifying therapies for OA, and existing pharmacological treatments for targeting symptoms are largely ineffective [3,4]. Nonpharmacological interventions such as exercise, weight management, and patient education have been shown to be effective in reducing OA-related pain and disability [5,6].

### OA Self-management

The latest clinical guidelines for OA recommend self-management, including health education and goal setting, as the core treatment alongside nonpharmacological and pharmacological treatments [7,8] when used as a supplement to medical care [9]. In the context of chronic disease management, goal setting and action planning have been found to improve self-efficacy, encourage behavioral change, and improve health outcomes [10-12]. Educational interventions, when delivered alongside exercise and weight loss programs, have also been shown to lead to better treatment adherence, reductions in pain, better self-management, and improved quality of life [13]. OA self-management tools can have an important impact on improving the delivery of interventional programs targeting patient education [14] and behavioral modification [15] and can also influence the effectiveness of patient–health care provider (HCP) communication and shared decision-making [9,16]. As such, acceptance and adoption of mobile health (mHealth) strategies for self-management can help improve health outcomes, reduce costs to the health care system, and encourage patients to take a more active role in improving their health.

### mHealth Apps

mHealth apps have shown promise in supporting patient self-management of health conditions, especially for chronic diseases that require long-term care and maintenance [9]. However, barriers to the adoption and use of mHealth technologies have been identified. First, the lack of user-centered designs integrating patients’ needs and preferences intercepts the adoption of and sustained engagement with such mHealth technologies [9,17]. Second, there has been a lack of formal mHealth evaluations to date [18,19], raising concerns about the safety and effectiveness of mHealth technologies [20,21]. Our understanding of the effectiveness of mHealth apps in supporting self-management and their impact on patient-reported outcomes in adults with knee OA remains in its early stages. Thus, there is both a need to fill the gap in the availability of mHealth apps for knee OA management and provide a framework to evaluate mHealth apps targeting OA self-management driven by the priorities and feedback of target users.

### Objectives

In our previous work, we explored the perspectives of end users on an mHealth app for knee OA self-management, leading to the co-design of an app prototype aimed at facilitating self-management and improving patient–physician communication [22,23]. The resulting app reflected a consensus of patient and HCP priority functional requirements, achieved through co-design. In this study, the overarching objective is to evaluate the app from both patient and HCP perspectives using qualitative and quantitative assessments. The primary aims of the 2-part patient evaluation are to assess the app by its (1) overall usability and quality, (2) ability to improve patient self-management behavior (goal setting and activity completion), and (3) effectiveness in improving quality of life, patient activation, and patient-HCP communication. The primary aim of the 1-time HCP evaluation is to assess the overall usability, quality, and perceived impact on patient-HCP communication.

## Methods

### Overview

We used a combination of qualitative and quantitative methods and triangulated data for increased rigor to evaluate the app features and functionalities from patient and HCP perspectives. Details on the main features and functionalities included in the app, which were determined in preceding co-design sessions, are summarized in Textbox 1. The app was assessed at baseline by patients and HCPs using semistructured interviews and surveys, followed by a 6-week usability period and a final follow-up survey evaluation for patients only. This study was reviewed and approved by the University of Calgary research ethics board (REB16-1372). Privacy and confidentiality of data were maintained throughout all phases of the study. All personal identifying information was stripped from interview documents and recruitment materials, and all the data were deidentified and stored in a password-protected electronic file on secure servers at the University of Calgary.

**Functionalities associated with each feature of the final mobile health app prototype.**

**Symptom tracking (dashboard)**

- Symptoms are tracked on the following dimensions: pain, stiffness, and functional limitation.
- Data on symptoms gathered from the patients were evaluated according to a threshold-based approach based on the Western Ontario and McMaster Universities Osteoarthritis Index criteria.

**Goals**

- Patients prospectively identify goals from a set list of categories (exercise and activity, pain reduction, and weight loss).
- Goals are further delineated into customized activities using the specific, measurable, attainable, relevant, and time-based goal-setting schema.
- Goals and activities can be tracked over a limited or continuous period.

**Activities**

- Activity is tracked on the following 2 dimensions: (1) activities related to goal setting (eg, linked to favorite activity, such as golf or biking) and (2) exercise (evidence-based recommendations from a physiotherapist or other health care provider)
- Activity categories provided were aerobic activity, aquatic activity, muscle strengthening, and others.

**Red flags**

- Red flags can be identified by patients in a journal-like manner, including any experienced symptom or activity-related difficulties that users may wish to discuss with their health care providers.
- Red flag categories provided were infection, trauma (eg, fracture), persistent inflammation, warmth, swelling or persistent pain, low mood, activity avoidance, or others.

**Resources**

- The summary of evidence-based self-management resources for knee osteoarthritis self-management was defined by 3 main headings: (1) information for self-management, (2) exercise therapies, and (3) guidance for goal setting.

### Patient Evaluation

#### Recruitment

Patients were recruited in partnership with the Arthritis Alliance of Canada and the Alberta Strategy for Patient-Oriented Research Support Unit (16/18, 89%) to capture a range of patient perspectives. We also recruited those who were included in preceding co-design sessions and expressed interest in participating (2/18, 11%). Adults aged ≥20 years with early knee OA and who experienced knee pain on most days of the month at any time in the past were eligible to participate. Patient participants completed a baseline (week 0) semistructured interview and survey evaluation, followed by a 6-week usability period and final (week 6) follow-up survey evaluation.

#### Qualitative Evaluation

Patients were provided with a weblink to the app 1 week before the launch of the baseline evaluation to allow them sufficient time to familiarize themselves with the main app features (dashboard and symptom tracking, goals, activities, red flags, and resources). At baseline, research team members (JM and ST) from the Patient and Community Engagement Research (PaCER) facilitated semistructured *talk-out-loud* interviews with patient participants, which served as an orientation to the app features and functionalities. The interview guide for the qualitative evaluation can be found in Multimedia Appendix 1. Interviews were conducted via telephone or in person depending on the patients’ preferences and were audio recorded for subsequent validation. The main objective of the baseline interview was to elicit patients’ initial impressions of the app related to user experience. Patient perspectives on the 5 main app features were elicited on the following parameters: (1) usefulness, (2) ease of use, (3) contribution to OA knowledge, (4) self-management potential, and (5) perceptions of the impact on patient-HCP communication. With the goal of summarizing the patient experience when using different app features [24], PaCER researchers subsequently used descriptive analysis to generate a compilation of descriptive statements that reflected the scope of responses for each of the 5 main app features. The descriptive statements were summarized using the following headings: likeability, usefulness, areas lacking and suggestions, and usability.

#### Quantitative Evaluation

Patient-reported measures related to app evaluation were collected using surveys administered at baseline and follow-up. The baseline survey collected information on patient demographics, history of OA symptoms and risk factors, quality of life, self-management behavior, and patient activation.

This baseline assessment was followed by a 6-week pilot trial in which patient metadata, including number of goals set and number of activities, were captured from direct inputs into the app and frequency of daily use. The follow-up survey collected information on perceived app usability and quality and reassessed postpilot quality of life and patient activation.

### HCP Evaluation

#### Recruitment

HCPs (n=7) were recruited through our primary care research partners, Enhancing Alberta Primary Care Research Networks and Accelerating Change Transformation Team (ACTT), using purposive sampling [25]. Enhancing Alberta Primary Care Research Networks and ACTT invited HCPs who represented the early majority, defined as the first sizable group of providers to adopt an innovation after it has been established by early adopters [26]. Participating HCPs included those who partook in the preceding co-design sessions (5/7, 71%) and those recruited via existing relationships with practices and primary care networks (2/7, 29%).

#### Qualitative and Quantitative Evaluations

The HCP assessment involved a 1-time evaluation comprising a semistructured interview and survey (see Multimedia Appendix 2 for the interview guide). During the interview component, 1 researcher from ACTT explored the app with the HCP and, using the *think-aloud* method [27,28], elicited their views on the clinical utility of the app, with emphasis on the perceived impact on patient-HCP communication. Interviews took place over the phone at a time of the HCP’s choosing, were approximately 45 minutes in length, and were audio recorded for subsequent analysis. The audio recordings were not transcribed but used later to validate the notes taken during the call. At the time of the interview, both the researcher and the HCP had access to the app to navigate its functions together. HCPs also completed a questionnaire to measure their perceptions of the app’s usability and quality.

### Evaluation Measures

Surveys were administered to patients at baseline and follow-up to evaluate changes in patient-reported outcomes over the course of the 6-week pilot trial. At the baseline patient evaluation, data were captured on demographics, knee symptoms, OA risk factors, quality of life, and patient activation. Quality of life was assessed using the European Quality-of-Life 5-Dimension 5-Level Questionnaire (EQ-5D-5L), a preference-based measure for describing and evaluating health, covering 5 dimensions (mobility, self-care, usual activities, pain or discomfort, and anxiety or depression) scored on a 5-point Likert scale [29]. The 10-item Patient Activation Measure (PAM-10) was administered to patients at week 0 and week 6 to provide a measure of patient activation, defined as a person’s knowledge, skills, and confidence related to managing their own health [30]. The PAM-10 was designed to minimize the response burden from its 13-item successor, the PAM-13, and has been reported to have comparable levels of consistency and reliability with the PAM-13 [31]. The PAM provides a measure of a patient’s engagement in self-management of their disease and thus enables change in patient activation to be monitored between baseline and follow-up evaluations.

At the patient follow-up evaluation, the EQ-5D-5L and PAM tools were re-administered along with additional measures assessing perceived app usability and quality. App usability was measured using the System Usability Scale (SUS), a well-established psychometric tool used worldwide with high levels of reported validity and reliability [32]. App quality was assessed using the App Chronic Disease Checklist (ACDC), an expert opinion–based checklist developed to evaluate the usability of chronic disease apps for monitoring, self-management, and behavioral change [33]. Finally, 2 sections (app subjective quality and app-specific quality) from the Mobile App Rating Scale (MARS) were used to provide an objective rating of perceived app quality [34], with each question measured on a 5-point Likert scale. However, it should be noted that not all sections from the ACDC and MARS tools were included in the assessment, as they included dimensions beyond the scope of the app prototype features (eg, gamification).

During the 1-time evaluation with HCPs, the SUS and MARS measures were used to assess perceived app usability and quality, respectively, from the provider perspective. Further details on each survey instrument used in the pilot testing evaluation are included in Tables 1 and 2.

**Table 1:** Description of measures used for qualitative evaluation of the app prototype throughout the 6-week pilot trial.

Measurement	Patients		HCPs^a^ at baseline	Method of elicitation
	Baseline	Follow-up		
Likeability, usefulness, areas lacking or suggestions, and usability	✓		✓	Semistructured *talk-out-loud* interview; see Multimedia Appendices 1 and 2 for patient and HCP qualitative interview guides, respectively, and Multimedia Appendices 3 and 4 for qualitative reports from qualitative interviews for patients and HCPs, respectively
Enhancing patient-HCP communication	✓		✓	Semistructured *talk-out-loud* interview; see Multimedia Appendices 1 and 2 for patient and HCP qualitative interview guides, respectively, and Multimedia Appendices 3 and 4 for qualitative reports from qualitative interviews for patients and HCPs, respectively
Increasing OA^b^ knowledge	✓			Semistructured *talk-out-loud* interview; see Multimedia Appendices 1 and 2 for patient and HCP qualitative interview guides, respectively, and Multimedia Appendices 3 and 4 for qualitative reports from qualitative interviews for patients and HCPs, respectively
Improving OA self-management	✓			Semistructured *talk-out-loud* interview; see Multimedia Appendices 1 and 2 for patient and HCP qualitative interview guides, respectively, and Multimedia Appendices 3 and 4 for qualitative reports from qualitative interviews for patients and HCPs, respectively

^a^HCP: health care provider.

^b^OA: osteoarthritis.

**Table 2:** Description of measures used for quantitative evaluation of the app prototype throughout the 6-week pilot trial.

Measurement and instrument		Patients		HCPs^a^ at baseline	Validated tool	Scoring methods	
		Baseline	Follow-up			Methods	Interpretation
**Quality of life**							
	EQ-5D-5L^b^	✓^c^	✓		✓	The EQ-5D-5L measures HRQoL^d^ on 5 dimensions (mobility, self-care, usual activities, pain or discomfort, and anxiety or depression). The health state of each participant is determined (series of 5 numbers corresponding to individual selections for each dimension) and then converted to an index score using a value set generated by and validated for the Canadian population.^e^	Index scores range from –0.15 to 0.95 using the Canadian value set, where low scores correspond to higher HRQoL, and high scores correspond to lower HRQoL; MIDs^f^, which is the minimum important change in EQ-5D-5L, scores are determined for specific patient populations and used to interpret EQ-5D-5L scores (MID for degenerative knee population=0.20).
**Patient activation**							
	PAM-10^g^	✓	✓		✓	A raw PAM score is calculated by summing responses for all 10 PAM questions for each respondent (scored on a 4-point Likert scale, where 1=nonactivated and 4=highly activated) and dividing the sum by the number of questions completed; mean PAM scores are converted to activation scores (scale from 0 to 100) using an empirically derived calibration table.	PAM score ≤47.0: people tend to be overwhelmed and unprepared to play an active role; they are predisposed to be passive recipients of care. PAM score 47.1-55.1: individuals lack knowledge and confidence for self-management. PAM score 55.2-67.0: people are beginning to take action but may still lack confidence and skills to support new behaviors. PAM score ≥67.1: people have confidence and perform adequate behaviors but may not be able to maintain them in the face of stress.^h^
**App usability**							
	SUS^i^		✓	✓	✓	For each of the 10 questions scored on a 5-point Likert scale, raw scores were obtained as follows: for odd-numbered questions, 1 was subtracted from the response value; for even-numbered questions, the response value was subtracted from 5; raw scores were converted to percentile ranks to map the raw SUS results to values calibrated from 446 studies, including >5000 individual SUS responses.	SUS score above 51 is interpreted as *okay,* with low marginal acceptability ranges; a SUS score >72 is considered acceptable, with *good* usability levels; and a SUS score >85 corresponds to *excellent* usability levels.^j^
**App quality**							
	MARS^k^		✓	✓		MARS provides an overall mean score (each question yields a score from 1 to 5) for different dimensions (quality, functionality, esthetics, and information) of a mobile app. Only specific sections were included; section E contains 6 questions scored on a 5-point Likert scale, whereas section F contains 4 questions scored on varying scales.	No official scoring mechanism used; reported response frequencies
	ACDC^l^		✓			A total of 14 questions are scored on a Likert scale from 1 to 3; only a subset of the relevant questions was extracted from the more comprehensive ACDC survey.	No official scoring mechanism used; reported response frequencies

^a^HCP: health care provider.

^b^EQ-5D-5L: European Quality-of-Life 5-Dimension 5-Level Questionnaire.

^c^Quantity evaluated.

^d^HRQoL: health-related quality of life.

^e^Bilbao et al [29].

^f^MID: minimal important difference.

^g^PAM-10: 10-item Patient Activation Measure.

^h^Titova et al [35].

^i^SUS: System Usability Scale.

^j^Bangor et al [36].

^k^MARS: Mobile App Rating Scale.

^l^ACDC: App Chronic Disease Checklist.

### Data Analysis

Descriptive statistics were computed for instruments measured only once at the baseline and follow-up assessments, including demographic characteristics, app usability (SUS), and app quality (MARS and ACDC), reporting the mean and SD or frequency and percentage where appropriate. Because of the small sample size, normality assumptions were not met; thus, nonparametric methods were used for all subsequent analyses. Wilcoxon matched-pairs signed-rank tests were performed to compare outcomes measured at both baseline and follow-up (PAM and EQ-5D-5L), including *P* values. In addition, app user metadata collected during the 6-week app usability period were exported, including the frequency of goal setting and activity completion. Spearman rank correlations (*r_s_*) were used to evaluate correlations between patient activation (as measured by the PAM), number of goals set, and number of activities completed for knee OA management, with correlations <0.20 considered negligible [37].

For the descriptive thematic analysis, PaCER researchers generated descriptive statements for each app feature that reflected the scope of the responses. Key messages were then extracted and summarized using inductive coding [38] (Multimedia Appendix 3). The notes taken during the HCP interviews were organized according to participants, questions, and app features. Using the audio recordings, the notes were then expanded upon and validated to ensure that they accurately represented the perspectives of participating HCPs. Once all interviews were completed, primary care researchers from ACTT compared similarities and differences in HCP responses and summarized the findings into descriptive categories by each app feature.

## Results

### Patient Evaluation

#### Qualitative Component

The main messages of the qualitative report generated by the research team members from PaCER (JM and ST) are summarized in Table 3. The corresponding detailed summary of the descriptive statements can be found in Multimedia Appendix 3.

**Table 3:** Summary of patient feedback from qualitative assessment at baseline (N=18).

App feature and areas of high likeability and usability			Areas lacking and suggestions for improvement		Implications for the patient–HCP^a^ visit
**Dashboard**					
	Provides a clear overall picture of the patient’s knee OA^b^ Creates visual prompts around issues that patients may want to discuss with their HCP, such as pain in relation to activity levels Likely to improve knowledge of OA—allows patients to moderate symptoms and identify limits	Should provide option for adding notes to symptom inputs Include reminders prompting patients to enter their symptoms and ability to enter data retrospectively Text should be enlarged for easier reading		Considered the feature that would be most likely to improve communication with HCPs—prompts patients to discuss issues such as pain and impact on activity levels Receptiveness of HCPs was considered a limiting factor	
**Goals**					
	Intuitive and simple to use Useful for encouraging patients to set goals (particularly for those who set goals infrequently); helpful for self-management	Goals were considered likely to work for activity and exercise, less so for symptom management Addition of reminders and a built-in reward system would be helpful Should include a notes feature so that patients can enter specifics on what was done to achieve goals Should link pain reduction goals to resources (less of a goal than an outcome)		Not considered a useful feature as patients do not typically discuss goals with their HCP	
**Activities**					
	Easy to update, plot, and review activities; liked the calendar view Helpful to remind patients to complete activities	Feature needs more specificity; should expand categories, for example, add duration to aerobic activities Present information as bullets and enlarge the font size More detailed instructions on how to use the activity feature Some overlap between activity categories (eg, aquatic and aerobic exercise) Link to exercise resources		Not identified as a useful feature for communication with HCPs	
**Flags**					
	Provides important visual link to activity avoidance Encourages patients to be more diligent and in tune with their symptoms Helpful for patients to look back on past flags and observe changes over time Helpful for self-management and avoiding or documenting acute episodes	Improve specificity of flags—expand the list of categories so they are more specific Add ≥1 descriptor per flag for improved specificity (eg, pain, low mood, and activity avoidance)		Helpful to note what patients would like to discuss with their HCPs Patients expressed that their HCPs may not be keen on using this feature Logging flags would add validity to the issues patients bring to their HCPs	
**Information**					
	Considered the best feature by most patients—particularly the exercise resources Good information from reliable resources	Include SMART^c^ goals link on the main page Include more strengthening exercises and guidance on exercise for specific patients (eg, with or without mobility issues)		Could help to encourage discussion of local resources and information that are relevant to specific issues	

^a^HCP: health care provider.

^b^OA: osteoarthritis.

^c^SMART: specific, measurable, attainable, relevant, time-based.

In terms of overall impressions, participants responded positively to the app, noting that it was simple to use and provided a complete picture of how things were going with their OA, along with a visual record to help them keep track of their self-management activities. Patients thought the app would motivate them to create goals, which in turn would encourage them to complete their activities. In addition, patients were keen on the *resources* feature, which included recommended exercises and other self-management information. Patients identified the dashboard as the most effective feature for improving communication with their HCPs, providing an overall picture of their knee OA and highlighting issues to discuss with their HCPs, such as pain in relation to activity levels.

However, participants expressed some hesitancy around the potential receptiveness of HCPs to the app, suggesting that this may limit the communication potential of the app. There was wide agreement among patients that the app would likely improve their knowledge of OA, enabling them to pay more attention to their symptoms and identify their limits. Patients emphasized that the *resource* feature would be most helpful and most frequently used, although those who reported having a greater understanding of OA were less confident that it would advance their knowledge of OA. Finally, most patients were hopeful that the app would help them self-manage their knee OA, and they were tentative to make predictions until they had used the app.

In the last phase of the qualitative evaluation, patients provided specific input on how a subsequent iteration of the app may be improved. Patients suggested that simplifying the data input function, making the app more personalized to individual goals, and enhancing the depth of content would be valuable enhancements (Table 3). Suggestions about making the app more user-friendly included more detailed instructions for each feature (including clickable information icons), expanding data input options (eg, allowing the addition of ≥1 activity or red flag per entry, a larger list of activity inputs, and retrospective data entry), and ensuring consistent terminology.

Recommendations from patients for improving the personalization of the app included the ability to add personal notes for activities and red flags. Feedback on enhancing the app content included adding a function to track pain medications, building in a reminder feature for activities, including more options and guidance on exercises for knee OA, and linking to more existing OA information tools (eg, My Health Alberta).

#### Quantitative Component

##### Demographic Characteristics

A total of 18 patients participated in the pilot trial, of whom 17 (94%) provided complete data at baseline and follow-up. The mean age of study participants was 62.2 (SD 6.9) years; 61% (11/18) of participants were female, and 83% (15/18) of participants had completed postsecondary education (Table 4).

**Table 4:** Baseline characteristics of patient participants in the app prototype evaluation (N=18).

Baseline characteristics				Values
**Demographics**				
	Age (years), mean (SD)		66.2 (6.9)	
	Sex (female), n (%)		11 (61)	
	Postsecondary education, n (%)		15 (83)	
**Knee symptoms**				
	Probable or definite diagnosis of osteoarthritis, n (%)		16 (89)	
	Experienced pain, aching, or discomfort in either knee for at least a month at any time in the past, n (%)		18 (100)	
	Number of days per month experienced pain, stiffness, or discomfort in either or both knees, mean (SD)		22 (12)	
	**Experienced any of the following symptoms in right knee, n (%)**			
		Warmth	10 (56)	
		Swelling	15 (83)	
		Redness	2 (11)	
		Inflammation	14 (78)	
	**Experienced any of the following symptoms in left knee, n (%)**			
		Warmth	7 (39)	
		Swelling	11 (61)	
		Redness	3 (17)	
		Inflammation	12 (67)	
**Risk factors, n (%)**				
	Engage in physical activity at least once a week		18 (100)	
	Have stopped or changed the type of physical activity because of knee pain		17 (94)	
	Have set a goal to improve KOA^a^ symptoms		10 (56)	
**Management of symptoms**				
	Have performed exercises or activities to improve KOA symptoms		14 (78)	
	Have ever set a goal to improve KOA symptoms		10 (56)	

^a^KOA: knee osteoarthritis.

##### Knee OA Symptoms and Risk Factors

All participants reported experiencing pain, aching, or discomfort in at least one knee within the past 12 months and reported experiencing pain, stiffness, or discomfort in either or both knees on an average of 22 (SD 11.5) days per month. Of the patients diagnosed with OA by a physician (18/18, 100%), 88% (16/18) received their diagnosis at least 1 year before participating in the study. All participants reported engaging in physical activity at least once a week, 94% (17/18) of whom reported stopping or changing the type of physical activity because of knee pain.

##### Knee OA Self-management

Approximately 78% (14/18) of participants indicated that they had performed exercises or activities to improve their knee OA symptoms at any point in the past, 67% (12/18) of whom had performed pain management activities or exercises >10 times in the past month. Regarding goal-setting behavior, 50% (10/18) of patients reported previously setting a goal to improve their KOA symptoms. Of those patients, 60% (6/10) reported setting at least one goal over the 6-week evaluation period, of whom 50% (3/6) indicated a success rate >50%.

##### Quality of Life and Patient Activation

Between the baseline and follow-up evaluations, patient activation, measured using the PAM, increased significantly from a mean of 80.4 (SD 9.1) to 87.9 (SD 9.7; Wilcoxon signed-rank test, *P*=.01). Patient quality of life, as measured by the EQ-5D-5L, changed from a mean of 0.77 (SD 0.13) to 0.67 (SD 0.26) between week 0 and week 6 evaluations, corresponding to a significant improvement in quality of life (Wilcoxon signed-rank test, *P*=.04; Table 5). The change in EQ-5D-5L score from baseline to follow-up exceeded the minimal important difference (MID) of 0.056 based on the Canadian population [39] but did not exceed the MID of 0.20 based on the Canadian degenerative knee disease population [40].

**Table 5:** Results of patient-reported outcome measures collected at baseline and follow-up evaluations by patient participants.

Outcome measure			Values, mean (SD)			*P* value
			Baseline (n=18)	Follow-up (n=17)		
**Quality of life**						
	**EQ-5D-5L^a^**					
		Index^b^	0.77 (0.13)	0.67 (0.26)	.04	
		VAS^c,d^	74.72 (19.36)	76.18 (17.64)	.45	
**Patient activation**						
	PAM-10^e,f^		80.4 (9.1)	87.9 (9.7)	.01	

^a^EQ-5D-5L: European Quality-of-Life 5-Dimension 5-Level Questionnaire.

^b^Low EQ-5D-5L index scores correspond to high quality of life (scale: −0.15 to 0.95).

^c^VAS: visual analog scale.

^d^High European Quality-of-Life 5-Dimension VAS scores correspond to high quality of life (scale: 0 to 100).

^e^PAM-10: 10-item Patient Activation Measure.

^f^High PAM-10 scores correspond to high patient activation (scale: 0 to 100).

##### App Quality and Usability

Following the 6-week pilot evaluation, patients were provided with a follow-up questionnaire to assess app usability and quality, quality of life, and patient activation. According to the responses to the ACDC assessing app quality, 53% (9/17) of patients with complete data reported that the app facilitated appropriate navigation, 65% (11/17) indicated that it was reasonably efficient, 71% (12/17) reported that it was user-friendly, and 88% (15/17) indicated that the app was free from confusing terms or jargon. Regarding the appropriate display of data and information, 88% (15/17) of patients reported that the app produced appropriate graphs or statistics for clinical data, and 77% (13/17) indicated that the app displayed correct and relevant information regarding their chronic condition and that visual explanations of concepts were clear, logical, and correct. In addition, the ACDC identified some areas of improvement for the app, with all patients indicating that the app had none or limited tactile, visual, or sound feedback, and 53% (9/17) reporting that the app did not facilitate ease of entering information. Patient responses to the MARS indicated an overall average rating of 3.1 out of 5 stars (rated on a scale of worst to best app ever used). As measured by the SUS, the mean score for perceived app usability was 57.8 (scale 0-100), indicating marginal usability according to SUS cutoffs [41].

#### Patient Metadata

During the 6-week observation period, user metadata were collected from the 18 participating patients, including symptoms of pain, stiffness and functional impairment, number of goals set, and number of activities completed toward knee OA self-management. The mean number of goals set during the usability period was 2.47 (SD 3.08), and the median was 2 (IQR 1.0-3.0). The mean and median number of activities completed for knee OA self-management during the study period were 22.2 (SD 17.8) and 18.0 (IQR 5.0-41.0), respectively. Spearman rank correlation (*r_s_*) calculations demonstrated that follow-up PAM scores were weakly correlated (*r_s_*=−0.32) with the number of goals achieved and the number (*r_s_*=0.19) of activities performed during the 6-week usability period.

### HCP Evaluation

#### Qualitative Component

A detailed summary of perceived likeability and usability, suggestions for improvement, and implications for HCP visits are summarized in Table 6 (see the detailed ACTT report in Multimedia Appendix 4). Overall, HCPs expressed support for the app features and functionalities, favoring the *Goals*, *Activities*, and *Resources* tabs. HCPs identified elements of each main app feature that would be useful for patient self-management and that could be useful in the context of a clinical visit. However, HCPs largely held the perspective that the app was too detailed and cumbersome for the patient population, typically comprising older individuals perceived as having limited technological literacy. Suggestions made by the physicians to make the app more user-friendly to this patient population included increasing the use of lay language to reduce high-level language and the reading level (grade 7 or lower; 3/7, 43%), increasing color contrast and using a colorblind-friendly palette (3/7, 43%), and providing areas to add notes or free text (3/7, 43%).

**Table 6:** Summary of health care provider (HCP) feedback from qualitative assessment (N=7).

App feature and areas of high likeability and usability		Suggestions for improvement	Implications for the patient-HCP visit
**Dashboard**			
	Helpful summary of symptoms and tracking of goals or activities Visual presentation of graphs and ability to track the completion of activities	The features of different events (ie, red flags and activities) could not be identified directly from the dashboard Add additional visual features for ease of reading for patients (eg, add a legend, increase contrast and font size, and reduce reading level)	Potentially too detailed to discuss within the scope of a patient visit—should be more usable at a glance Might consider highlighting pain as a main source of discussion during the patient visit (stiffness and functional impairment are less relevant)
**Goals**			
	Use of the SMART^a^ goal-setting framework Incorporation of an assessment of confidence in achieving goals (ie, “how confident are you that you will be able to complete this goal?”)—marked on a 5-point Likert scale from not confident (1) to very confident (5) Summary of goals and prompts for next scheduled activity is useful	There could be more clarity on how to use the feature—HCPs thought it might be too complex for patients to follow Improve visuals for easier reading—increase font size and color contrast Too many categories of goals Achieved goals should be removed Keep the page to one screen so that scrolling is not required	Feature is most relevant for self-management
**Activities**			
	User-friendly and straightforward data entry	Modify activity categories from drop-down list—make it more relatable for those who are less exercise-oriented Provide definitions for activity categories List of activity categories is limited, and language is too high level Improve readability using color contrast and different coloring	Need to include an option to go back to completed activities to discuss with HCP Would be useful to see a percentage of activities completed
**Flags**			
	Helpful for capturing activity avoidance	Suggestions for updates or modifications to the red flags list were provided Add option to provide notes to accompany a red flag Add option to highlight red flags to be discussed with HCP	Add option to highlight red flags intended to be discussed with an HCP
**Information**			
	Useful feature; considered the best tab by most physicians Information on exercise therapies was simple to understand with appropriate images and videos Reference to evidence-based programs (eg, GLAD Canada) and no equipment requirement Printable format ideal for older patients	Provide exercise adaptations for patients who may be mobility-limited Provide more local resources and guidelines Add a frequently asked questions section Separate information page for resources for patients and resources for HCPs	Feature is most relevant for self-management

^a^SMART: specific, measurable, attainable, relevant, time-based.

#### Quantitative Component

##### App Quality and Usability

During a 1-time app evaluation, HCPs (n=7) completed a questionnaire assessing app usability and quality from the patient perspective. According to the responses to the MARS, 71% (5/7) of respondents agreed that the app was likely to increase knowledge of knee OA, 86% (6/7) of respondents indicated that the app was likely to increase motivation to address knee OA, and 71% (5/7) of respondents considered that the app was likely to improve self-management practices. When asked if they would recommend the app to patients with OA who might benefit from it, 57% (4/7) of HCPs responded affirmatively. Overall, HCPs rated the app as 3.0 on a scale of 1.0-5.0, indicating the potential for improvement. As measured by the SUS, the mean score for perceived app usability was 39.1, pertaining to unacceptable usability according to SUS cutoffs.

## Discussion

### Principal Findings

We have demonstrated that an app prototype co-designed with end users has the potential to successfully deliver self-management guidance to patients with knee OA. During the 6-week observation period, patients experienced significant improvements in patient-reported quality of life and patient activation and exhibited high levels of engagement with the app, as demonstrated by a high number of activities completed and goals achieved during the usability period. However, usability scores were reported to be in the marginal range for patients and an unacceptable range for HCPs. The overall average app ratings from patients and HCPs demonstrated the potential opportunity for further quality improvement.

The qualitative assessment revealed that, from the patient perspective, the app features, including the dashboard, goals, activities, flags, and resources, were useful and user-friendly but could be expanded to include functions that are more personalized and specific to each patient’s lived experiences, such as the ability to add notes and reminders. Patients indicated that they were most likely to use the *resources* feature, emphasizing that the knowledge aspect of the tool was important to their self-management. Furthermore, patients largely viewed the app as a catalyst to increased autonomy in self-management. Many elements of the app features were viewed as useful by HCPs, particularly those related to the inclusion of evidence-based information; specific, measurable, attainable, relevant, time-based goal setting, and exercise therapies for OA.

However, there were gaps between patients’ and HCPs’ perceptions of app usability and quality, with HCPs expressing greater concern than patients about the patient’s ability to effectively navigate and use the app features. This is consistent with our previous work [22], where physicians and patients expressed different views on the seriousness of knee OA and their approach to its management and also their perceptions of whether those diagnosed with knee OA can manage their disease using an app or other mHealth tools. Despite patient receptiveness to the prospect of using an mHealth tool such as an app, patients and physicians held diverging views, where physicians were concerned about the technological literacy of the conventionally older OA population. Negotiating consensus during co-design about the app features beneficial to both patients and physicians may help align patient and physician perspectives. Furthermore, improved patient-HCP communication and the discussion of priorities and best-available evidence may help bolster shared decision-making [42].

### Limitations

We recognize several limitations of our study design. The evaluation sample size was small but aligned with the suggestion by Nielsen et al [43] that for projects of medium to large size, 15-20 test users are optimal to balance evaluation costs with the benefits of finding usability problems in testing. Furthermore, our patients were predominantly female and had higher educational attainment. Thus, our patient sample may not be representative of the target early knee OA population, as research has shown that active participants research may have different motivations and priorities compared with those who are less engaged [44], and departures from representativeness may be amplified with increasing age [45]. This may be reflected by our study findings, in which 88% (15/17) and 100% (17/17) of participants reported PAM scores in the highest range of patient activation at baseline and follow-up assessments, respectively, despite the use of multiple recruitment mechanisms intended to capture a range of patient perspectives.

Similarly, the HCPs involved in the design and evaluation of the mHealth app were primarily family physicians. Thus, our findings may not reflect the diversity of perspectives among HCPs that are typically involved in guiding self-management for patients with knee OA. Further studies will expand the focus of the evaluation to assess its applicability in a more diverse group of HCPs (eg, physiotherapists). In addition, the inclusion of patient partners with a wider range of experiences related to education, health literacy, and technological proficiency in future app development and evaluation is essential to address potential health inequities [46].

There were shortcomings in app usability, as demonstrated by the low SUS scores reported by both patients and HCPs. However, usability challenges were of greater concern among HCPs in comparison with patients who reported higher usability scores and identified minimal to no usability challenges in the qualitative assessment. This discrepancy between patient and HCP perceptions of usability is aligned with the findings from our preliminary work [22], where HCPs largely underestimated patients’ receptiveness and ability to use mHealth technologies for OA self-management. It is possible that HCPs evaluated the app usability with a broader OA patient population in mind than that represented in our sample, who were both highly educated and demonstrated high levels of activation in their care.

This study had a relatively short duration of follow-up of 6 weeks. As such, the emphasis on the evaluation of patient-reported outcomes against clinical outcomes was suitable, as it was unlikely that we would observe clinically meaningful changes in OA symptoms during the short observation period. However, although significant improvements in health-related quality of life and patient activation were reported during the study period, the change in EQ-5D-5L scores did not surpass the MID established in the degenerative knee population. Thus, the length of follow-up may have been insufficient to establish meaningful changes in some patient-reported outcomes. Given the chronic nature of OA, using a longer evaluation period for a future iteration of the app would provide more relevant data on clinical outcomes to augment the patient-reported outcomes featured here and additional context for whether long-term engagement with the app could be sustained. Further app development involving an evaluation that is more inclusive of diverse patient and HCP perspectives, with a longer follow-up duration and a wider range of outcomes measured, will be an essential next step in this study. In addition, we are exploring opportunities to integrate self-monitoring data with advanced machine learning analytics to provide an intelligent platform to assist patients with knee OA in initiating and sustaining self-management activities.

### Comparison With Previous Studies

To our knowledge, this is the first mHealth app developed specifically to aid in knee OA self-management, although several digital self-management programs have been developed for OA [47] and other chronic diseases, including diabetes [48,49] and heart failure [50]. A 2020 systematic review on digital health interventions for people with OA identified that most (5/8, 63%) studies were primarily focused on health education (n=5), whereas some incorporated additional self-management elements, such as goal setting (n=6), action planning (n=4), physical activity (n=6), weight management (n=5), and pain management (n=6) [47]. A 2017 systematic review focusing specifically on mHealth technologies identified a lack of emphasis on tracking OA symptoms and self-management behavior that could be useful for shared decision-making [9]. The review by Choi et al [9] provided a framework for developing mHealth apps for OA management, describing the need for patient-facing mobile apps with capabilities such as symptom monitoring, activity monitoring, joint function measurement, physical activity guidelines, educational content, and provision of data visualization and summary reports for shared-decision-making. Thus, our app addressed many of the identified market gaps for apps in the OA self-management domain by incorporating capabilities that have been established as integral to facilitating self-management, decision support, and shared decision-making.

Regarding methods for evaluating mHealth tools, a recent scoping review summarizing quality assessment methods for mobile apps in chronic disease management found minimal agreement on the most appropriate criteria for evaluating mobile apps, with only 18% (12/65) of apps including evidence-based health information, 22% (14/65) using a behavioral change framework, and 37% (24/65) applying usability metrics as quality criteria for app assessment [51]. On the basis of the gaps identified in the analysis and practice methods for evaluating mobile apps, the authors proposed 3 primary goals for building quality criteria for app assessment: (1) prioritize existing evidence and knowledge against ease of assessment (eg, using patient-reported outcomes vs app ratings), (2) emphasize principles of behavior change theory, and (3) explicitly incorporate the patient perspective. Our study begins to address these identified gaps in quality assessment methods using a range of evidence-based patient-reported outcomes, incorporating behavioral change principles such as goal setting and activity monitoring, and integrating the patient perspective from early planning to app assessment phases.

### Conclusions

This pilot study provides support for the use of evidence-based, patient-centered mHealth apps to improve patient-reported outcomes by encouraging patient self-efficacy and improving patient-HCP communication, ultimately promoting conservative management of knee OA. These findings, along with findings from previous study phases, provide a framework from which app developers and researchers can co-design and evaluate mHealth apps targeting self-management in a way that is inclusive of all stakeholders and reflects diverse user perspectives. Further development of the app to address usability and feasibility in the context of a larger evaluation trial, including a more diverse and representative population sample and a longer period of evaluation, will be instrumental in understanding the impact of the app on self-management and a broader range of clinical outcomes such as pain and disability. In addition, our findings can provide a basis for developing best-practice reporting standards and practices to evaluate evidence-based mHealth technologies that target self-management of chronic conditions. Future app development may involve the integration of machine learning to provide personalized self-management recommendations to patients with knee OA to address individual needs and priorities.

## Appendix

Multimedia Appendix 1 Pilot testing interview guide for patients.

Multimedia Appendix 2 Pilot testing interview guide for physicians.

Multimedia Appendix 3 Descriptive statements from the patient qualitative evaluation (n=18).

Multimedia Appendix 4 Accelerating change transformation team health care provider evaluation summary (n=7).

## Abbreviations

ACDC: App Chronic Disease Checklist
ACTT: Accelerating Change Transformation Team
EQ-5D-5L: European Quality-of-Life 5-Dimension 5-Level Questionnaire
HCP: health care provider
MARS: Mobile App Rating Scale
mHealth: mobile health
MID: minimal important difference
OA: osteoarthritis
PaCER: Patient and Community Engagement Research
PAM: patient activation measure
SUS: System Usability Scale
