# Supplementary material for: Patients’ Use of Mobile Health for Self-management of Knee Osteoarthritis: Results of a 6-Week Pilot Study
Source: JMIR Form Res. 2021 Nov 25;5(11):e30495. doi: 10.2196/30495 (PMC8663438; doi:10.2196/30495)
Supplement: Multimedia Appendix 1 [file formative_v5i11e30495_app1.docx]

**Appendix 1: Pilot testing interview guide for patients**

These probes act as a general interview guide for the interviewer.

*Introduce and frame the question*: We have provided you with a link to the KOASK App Minimum Viable Product (MVP), which is the product of multiple interviews and co-design sessions with family physicians, early knee osteoarthritis patients, and researchers. The MVP represents a balance of patient- and physician-identified priorities, budget, and timeline. The tool is to be used as a self-management tool by patients. In addition, patients may also bring the App to their physician visits, therefore we are seeking to understand your initial impression of the App’s usability, quality, and impact on the patient-physician visit. This is only the first iteration of this App, so it is valuable to gain your perspective in order to inform future versions of the App as we pursue further grant funding.

I would like to talk through the App with you, and have you provide your immediate reactions and feedback. Following the talk-aloud we require you to complete a short questionnaire (either the interviewer can complete this for you or you can complete it on your own). Lastly, we would like to ask your thoughts on which features you think strengthen or do not strengthen patient-physician communication about KOA.

**Probes for interviewers: impact of features on patient-physician communication**

1. I am going to walk you through the five tabs, each which represent one of the main App features. Tell me what you think about this feature? *(repeat for Dashboard, Goals, Activities, Flags, Resources)*

- What do you like about this feature? What do you not like about this feature? Why?
- What do find useful about this feature? What don’t you find useful about this feature? Why?
- Is there anything that you think this feature is lacking or would change? Why?
- Were there any features that you found difficult to use? Why?

1. Of what we have reviewed so far, what feature do you think would most improve communication during visits with your health care provider (s)?
2. Did you think the App was easy to use?

- Why or why not?

1. Do you think the App is likely to increase your knowledge/understanding of knee osteoarthritis?

- Why or why not?

1. Do you think that the App is likely to improve your self-management of your knee osteoarthritis (frequency/length of activities, goal setting, etc.)?

- Why or why not?
