# Supplementary material for: Patients’ Use of Mobile Health for Self-management of Knee Osteoarthritis: Results of a 6-Week Pilot Study
Source: JMIR Form Res. 2021 Nov 25;5(11):e30495. doi: 10.2196/30495 (PMC8663438; doi:10.2196/30495)
Supplement: Multimedia Appendix 3 [file formative_v5i11e30495_app3.docx]

**Appendix 3:** Descriptive Statements from patient qualitative evaluation (n=18)

|  | Dashboard | Goals | Activities | Red Flags | Resources |
| --- | --- | --- | --- | --- | --- |
| **Likeability** | Overall participants thought this feature looked clear and useful.  They liked that it:  - provided a complete picture,  -a visual record that will help them keep track of what they are doing & how things are going  _________________________  Some participants found it:  -unappealing and bland  -the font in the instructional links was too small. | Overall participants thought this feature looked good.  Prompting them to read the info re SMART goals is good.  One person who doesn’t set goals thought this would prompt her to do so  _________________  Participants indicated had not used this feature enough to identify things they didn’t like about it. | Overall interviewees like the look of this feature and thought it would be easy to use.  They liked:  -being able to update activities;  -can see & plot activities for the month;  -can check specific days.  _________________  Needs to be expanded and more specific, for example they should be able to type in more than one activity including its duration.  Instructions could be improved, for example, when you click on an actual date there needs to be more explanation on how to mark that one.  Information should be presented as bullets and font larger. | Overall participants liked this feature.  Would help them keep track of what to discuss with their doctors. (However, suggestion that doctors may not be all that keen on this.)  Would provide evidence of why you weren’t doing activities.  _________________  Could be more specific, for example, while you can choose ‘other’ as a flag you can’t put what it is.  The flag on the dashboard should specify the problem.  Drop down menu hard to use – it turned out to be a Mac issue. | Quite a bit of enthusiasm about this feature  Unlike other features, they had actually tried some of the exercises.  Thought websites had good information from reliable resources.  The look forward to linking to these resources.  _________________The ease of use was a problem for some as some of the links didn’t work. |
|  | Dashboard | Goals | Activities | Red Flags | Resources |
| **Usefulness** | Useful in these ways:  -shows where they stand  -it will show trends/patterns over time.  - Would get one participant thinking more about how he is actually functioning.  _________________________One person not sure how knowing the classification of his pain (moderate) would actually help him,  One thought seeing his OA as it really is might be more depressing than actually living with it day-to-day.  Wondered where personal notes could be added, e.g. why they would stiffer than usual on a particular day. | Though feature looked useful as:  - it would encourage them to create goals, giving them a push to use them.  -Seeing it in writing will remind them they have goals.  _________________Participants indicated had not used this feature enough to identify things that weren’t useful.  Thought the goal feature work better for the activity and exercise features than for the pain and function features. | Some thought this feature would likely be useful as:  - you’d be able to see how you are doing at a glance  -it would remind them to do their activities. _________________Some weren’t convinced of the usefulness of this feature as:  - they found it confusing (e.g. a particular activity would also fit under aerobic activity).  -activities needed more specificity and should be linked back to goals. | Participants thought the flag options were useful (e.g. activity avoidance).  Flags would make one person more diligent at working to overcome it (e.g. activity avoidance)  Over time they’d be able to look back and find what had gone wrong on a certain day.  __________________  Some reservations about usefulness:  -didn’t know why these specific categories were chosen  -categories weren’t intuitive or exhaustive (e.g. inflammation and joint pain are part of OA unsure why that would be a red flag) so one wondered  Want to be able to activate more than one flag at a time: pain, activity avoidance, low mood. | Participants thought this was good practical information, particularly related to exercises, and goal setting resources.  _________________  In the mind tools resource you get jus 3 free articles then you have to pay.  Some found the exercise information less useful as they already do them. |
|  | Dashboard | Goals | Activities | Red Flags | Resources |
| **Lacking/**  **suggestion** | Some comments seemed to be suggesting that the app doesn’t tell the whole story (for a person):  -putting a name on the pain doesn’t help manage it.  -knowing function is rated as “OK” doesn’t help deal with the fact it is hard to do things, e.g walking down the stairs.  Suggestions:  -Add a place to track pain medications  -Rename ‘help’ link to ‘more information’ as would be more inclined to click on the latter  -Add a feature that reminds a person to input their data  -Date scale should state the date vs using numbers 1 to 30  Allow for entering data from past dates | Suggestions:  -Build in an activity reminder  -Build in a reward system  -Give a more detailed description on the benefits of exercise  -Create a pop-up that asks “did you know?”  -Separate duration and intensity  -Need to be able to add what did specifically | Feature not specific or descriptive enough to the individual.  You need to be able enter personal details, (e.g. overdid exercise one day and was stiff and sore the next).  Need to be able to enter all the specific activities you do each day, (e.g. as with Garmin Vivo Fit.  Suggestions:  -Enable linking to other apps such as Garmin  -Link directly to the resources for this feature: one person said this was using the principle “most teachable moment” (412). | Need to be able to specify what the flag means, not just show a flag, (e.g when go to doctor it’snot helpful if 5 flags show on the dashboard without any information.  Categories should be more specific, or likely better yet) individually defined/described.  On Garmin Vivo Fit you can enter different activities and see them all on the app. | Suggestions:  -Include a way to keep track of medications  -Put the link to SMART goals at the front rather than burying it in resources: to make goal setting less intimidating  -Re exercise: include more strengthening vs so many stretching  -Give guidance on which exercises to use  -Use My Health Alberta for goal setting & losing weight |
|  | Dashboard | Goals | Activities | Red Flags | Resources |
| **Usability** | Participants thought the app was/would be easy to use:  -the set-up is good  -there aren’t a lot of “clicks”,  -doesn’t seem to take too much time.  Person who has problems with both knees could enter information for just one  Measurement scale would mean something to professionals but not necessarily to patients: One person called the scales a ‘mystery’ | ______________  Provide a link on how to craft a pain reduction goal.  Consider pain reduction is an outcome rather than a goal. | _________________  Hard to use as categories are too broad, e.g. is biking an aerobic exercise or a muscle building.  Icons need to be congruent/consistent, e.g. icon of a man running to depict activity is different in the top navigation and goal is depicted as both a trophy and a star. | _______________  The generic nature of the flags and the fact you can’t type an item into the ‘other’ category made this feature hard to use. | ­­  ________________  A disconnect between ‘information for self-management’ and ‘knowledge’: use just one term.  Are all resources accessible in Alberta. |
