# Supplementary material for: Patients’ Use of Mobile Health for Self-management of Knee Osteoarthritis: Results of a 6-Week Pilot Study
Source: JMIR Form Res. 2021 Nov 25;5(11):e30495. doi: 10.2196/30495 (PMC8663438; doi:10.2196/30495)
Supplement: Multimedia Appendix 4 [file formative_v5i11e30495_app4.docx]

**Appendix 4: Accelerating Change Transformation Team (ACTT) health care provider evaluation summary (n=7)**

**DASHBOARD**

All participants liked the score/numbers on the bottom of scale and thought that the Colours & icons were clear and user friendly. One participant commented that they assumed the colours were ‘ok for people who are colour blind’. The visual presentation of the graph, i.e. month view and month worth of data, and the ability to track when activity had been completed was also viewed as helpful by the majority.

Items that participants did not like were the "Scale/date itself in that it might be too small if someone has vision problems. All participants commented on the use of a scale of 0-100, they stated their preference would be to use 1-10. One participant commented that the colors used (red black & blue) didn’t make sense. In addition, one participant commented that the measurement of Pain Stiffness & Function (PSF) was counter intuitive, mainly because on the graph when data is entered it looks as if functioning is improving but in fact its impairment in functioning that is getting worse so a higher score indicates an increase in suffering. Function is in fact an impairment score. Participants liked that patients could identify goals but their addition to this graph makes it very busy with too much data. The Scale/axis is not labeled and when you click on the red flags they don’t say what it was that the patient experienced to create a red flag i.e. what activity was it that they did, what goals were set on what date? It was also suggested that the position of the red flags was not aligned and should be at the top or bottom of the page and not on the graph itself. Two participants found the dashboard very confusing.

Features that some participants found difficult to use included: "The screen being white, it wasn’t clear on the dashboard where to record activities". One participant commented that they spent an hour trying to figure out how to use this app feature and they consider themselves computer literate, adding most seniors are not. In regards to monitoring stiffness one participant said that they had never asked a patient this, as most would report morning stiffness as a symptom of OA.

Suggestions for improving the Dashboard tab were “This needs to be a communication tool that requires a quick glance so that Dr. can deduct quickly but it takes time to figure out each item and is time consuming and not helpful in the patient visit” also, “Needs a legend Change and grid patterns should use darker lines”. One participant commented on the classification of Pain Stiffness and Function stating that “Most patient’s goals are to control pain symptoms and be able to do things they want to do not sure how useful stiffness & function would be” They also recommended changing the font size to make it larger and reducing the reading level to grade 7. Another participant suggested adding a legend and the ability to scroll through and see what activities were completed on what days rather than click through. Add a link to definitions of pain stiffness and function, showing good function vs bad function. Red flags and PSF are currently floating above the X axis – move closer.

**GOALS**

Participants liked that it automatically populates the statement of actions. Great feature which gives the patient accountability for setting goals and how they are going to achieve them. Two participants commented that following SMART goals with the inclusion of confidence is significant to success. Participants also liked how the patient can set their own goals. Seeing the next activity that the patient is scheduled to complete was useful.

Although clear one participant commented that this tab was too busy, with too many categories. One participant didn’t like much about this tab commented that this tab was plain and not very clear and too busy on the lower part.

All commented on the exercise & activity items that were listed multiple times, asking “Why so many categories”? One participant tried to delete some activities and another commented that it “Doesn’t say when created on – there are no dates on this page, “another added the actions made no sense. The font was described as too small and to some it was not clear if they were seeing one goal in each area. One participant was not sure if the patients make the goals alone or with physician, also were not sure if this page links to the dashboard. Having to scroll through all the items and not being on one page was considered too much for a senior patient. One participant shared that this tab doesn’t help much, and asked what it is all about?

Suggestions for adding: Make it visual and present in a different way. Have buttons on each activity. Keep the page to one screen so you don’t have to scroll down. Have one button for each category. When the goal is achieved it should be removed from the page. Set one goal in each area then when achieved then allow the user to set another goal. Only have one click for exercise and activity instead of multiple entries? “You have to use your brain to interpret what to do – the tab is lacking context” need to add more context to this page. Take out red & yellow colors and use different shades of green (traffic light of warnings effect) the current use of colours can trigger some patients.

**ACTIVITIES**

Generally, the participants agreed that it was straightforward to enter data on this tab. That its specifically about exercise provides a nice snap shop. This is tab was described by one participant as the best tab very clear, easy to use.

Features that participants found difficult included the layout and colours, they need more of a contrast to make it easier to read. The list of categories is too limited and would not mean much to most patients. All participants questioned the use of categories e.g. “Why were Aquatic and Aerobic used”? Categories need to be better defined for patients or people who don’t exercise. e.g. swimming, walking or gardening, more relevant activities to this population. In the option ‘other’ there is no drop-down menu or area for patients to document – they won’t remember when they come to the appointment. One participant commented that the tab was depersonalized, and the font too small. One participant commented that the drop-down menu “doesn’t really tell me what I did” and was not sure of the point of it. Links not working so this made it difficult to evaluate. One participant commented that it seems you just enter data; this page isn’t for obtaining information because there is no record of what you have done? You can’t look back.

Suggestions: Change the categories aquatic & aerobic to more layman terms, language too high level and fancy, and give examples for this specific population using language that patients would recognize. Some participants suggested recording aerobic activity, what type and for how long. One participant suggested it would good to see the % of activity completed. Have free text area take or leave ‘other’ out. Writing the exercise goal here would be a good prompt to remind people. Add a green check mark when an activity is achieved. Look at Exercise is Medicine Canada website - they have definitions for activities, it was suggested the research team refer to these. Set up as a table to capture the readers attention people like to write on paper.

**FLAGS**

Comments related to this tab centred around there being no space to complete ‘other’ and the same issue with the drop-down menu and nowhere to record any concerns. One participant felt that the content was helpful for a doctor and captured activity avoidance, a Simple page. Another participant commented that the categories do not make sense e.g. infection too vague, trauma – not sure why this is included? There was concern that the categories listed some of them warranted seeing a Dr. e.g. Infection, low mood category. There should be a statement that patients should make an appointment with their Dr. if they have signs of infection. One participant noticed that Inflammation is spelled incorrectly and suggested that the developers use e.g. instead of ex.

One participant commented on the categories warm persistent pain and asked what if its only one of those? They also noticed that Pain, Swelling and Function were not listed as it was on the dashboard. The categories and making the patient choose between them is likely not effective. Any of the categories stated will result in avoidance of exercise. Should allow the choice of multiple. Not every patient would use the persistent pain category.

Participants commented that the reading level too high for most patients. There is a need to define language in more layman terms e.g. trauma, inflammation. What is the difference between warmth/swelling/ persistent pain & inflammation? One participant commented that there is a lot of guess work involved here because not sure if this links to the dashboard so a lot of back and forth between the two tabs. Another noticed that the exact same calendar as previous tab with the same shade of blue, they suggested changing the shade to a different color. One participant suggested giving examples of what might be a red flag and have a way of highlighting that I will speak to my Dr. about this or what to speak to Dr. about if a flag is selected. More useful to record are you having extreme pain or different degrees of pain, are you having a bad day? Change inflammation to an increase in redness and swelling Have this in a box. Change boxes to red to match red flag color Add a free text box Change language in drop down menu – change trauma to injury, inflammation to swelling

Change the order in the drop-down menu to Low mood to pain, change trauma to injury which is more frequent than trauma, list swelling. Should be able to scroll instead of clicking all the time Not sure this would be user friendly if used on a cell phone. Create a space for someone to diary. People like to take notes/write things down

**EXERCISES**

This was described by some participants as the best tab for physicians. Participants liked the pictures showing the exercise therapies & videos links, but some felt that the exercises are beyond a lot of this patient population abilities, not many patients would be able to perform exercises on their own. Participants were pleased the GLAD program is included. Colours were considered good for patients who may be colour blind. Some liked that its kept to 6 simple exercises. Good that no equipment is included in the 3-6 simple exercises. Participants liked that you can print this page and there is good use of white space so that one could write on the print out. Appropriate groupings but one participant shared that one of the videos has a bendy flexible girl and would not relevant to seniors and will not encourage then that they can perform the exercise. In the actual exercise therapies, it was suggested that researchers add a link to the GLAD program. Also add a myth buster section what is true about what you can or cannot do with OA and provide the rationale – there are a lot of misconceptions <http://www.cfpc.ca/uploadedFiles/CPD/OATOOL_FINAL_Sept14_ENG.pdf>

Review exercise therapies and make it more appropriate for the population you are trying to reach. Exercise therapies – as adaptive exercises (chair or exercises for able bodied and less able-bodied persons). Always provide links to videos for people to watch the exercise being performed and make the exercises more relatable show someone holding onto a chair and stretching.

**GOAL SETTING**

Participants liked that the topic is included as often its ignored because it’s challenging for most patients. Good that it’s a 1- pager. Liked the links, broad concept and good summary. One participant felt that there was too much information, quite overwhelming and busy Seniors would need someone to go through the goal setting with them. You need some level of education to complete the goal setting exercise. Another added that it would be useful to add SMART goals explanations.

**INFORMATION**

The overall feedback for this section was that there were too many resources, suggested that you only give 1-2 not 10 because people get confused. Diagnosing is more for HCP not for patients. The GLAD feature and 811 link not helpful for self-management – nothing to do with OA Your Treatment – this is not a patient resource there is a complete list of drugs for OA that may not be relevant for patients.

Separate this page into for patients and for physicians. Keep the patients page very easy, specific and with relevant links <http://www.cfpc.ca/uploadedFiles/CPD/OATOOL_FINAL_Sept14_ENG.pdf> One participant added “This tab really needs an overview from the patients perspective and what would be useful to them e.g. What can I do about my pain? What are my local resources? How can I access the local resources?”

The GLAD should link directly to their resource on OA & your treatment. Current GLAD link not relevant, too general about GLAD. Add descriptors of all links e.g. this is a program you can look for etc.

Keep the resources local, Canadian context, even just the AB context. International guidelines and treatments differ from what happened in Canada, even provincially.

One participant wasn’t sure what you want them to get from the numerous websites. Another suggested adding something for patients such as FAQs e.g. when is a referral for a joint replacement relevant. Stick to picking 4 websites maximum allowing user to go to individual sites. Keep the resources to the local context & Canadian and consider customizing to the arthritis society guidelines.

**GENERAL FEEDBACK ABOUT THE APP USAGE (Qs 2-5)**

If you don’t use the back arrows you are kicked out of the app each time – provide instructions to avoid this happening

Links didn’t work so made it difficult to fully evaluate the tabs

Change the order of the tabs and have goal setting as the first tab.

Good having graph as a visual to use in the patient consultation

Seniors like to write things down – there is nowhere to do this.

Only have 15 minutes with a patient in its current format this app would take more time than the visit allows to review

Likely wouldn’t refer to it in its current state

App at this point likely more useful between a patient and someone that has more time to spend with them i.e. exercise specialist

Would be better to have a 1 pager for a physician to glance at rather than having to click on tabs – maybe the dashboard is intended for that?

Having the resources for patients and physicians in one place is useful

Add directions in the app e.g. type in box 1 and box 2 etc.

Add more prompts for the user and be more directive in what they need to do

Focus on pain & functional ability e.g. what are they able to now and what they would like to do

Add medications – what is their current pain medication? Are they using herbal or alternative remedies? how many anti-inflammatory drugs are they taking? Is pain a barrier to their ability to do what they want?

Add in the ability for the patient to personalize the app – add name, weight, age etc. baseline recordings Add the ability for the patient to print or send graph to email

Use a mood scale to record mood otherwise there doesn’t appear to be a way to record mood which can affect motivation

Improve its functionality as mentioned before and make into an android or iOS app

Not sure about the age group with KOA and if they
